# Damage‐Free Full‐Thickness Dicing of Ultra‐Thin GaAs Wafers Using a Femtosecond Laser with Low Residual Stress

**DOI:** 10.1002/advs.202515347

**Published:** 2025-11-19

**Authors:** Shunshuo Cai, Yankang Ding, Minxia Ding, Qi Song, Zhe Zhang, Siwei Zhang, Kunpeng Zhang, Yu Hou, Song Yue, Haiyan Shi, Man Li, Wenrui Duan, Zichen Zhang

**Affiliations:** ^1^ Microelectronics Instruments and Equipment R&D Center Institute of Microelectronics Chinese Academy of Sciences Beijing 100029 China; ^2^ School of Instrument Science and Opto Electronics Engineering Beijing Information Science & Technology University Beijing 100192 China; ^3^ School of Integrated Circuit Science and Engineering Beihang University Beijing 100191 China

**Keywords:** chipping‐free, femtosecond laser, full‐thickness cutting, GaAs, ultra‐thin

## Abstract

Gallium arsenide (GaAs) is a widely used semiconductor material due to its low‐temperature coefficient and high absorption efficiency. However, its hardness and brittleness create challenges in wafer‐level packaging, especially for large‐size and ultra‐thin GaAs wafers. These challenges include chipping and the formation of a wide heat‐affected zone (HAZ), both of which reduce production yield. Here, a dicing method is proposed that utilizes a non‐diffracting Bessel beam to shape the femtosecond laser, enabling high‐speed, high‐precision, and high‐aspect‐ratio dicing of brittle GaAs wafers while avoiding the thermal damage and debris issues inherent in conventional methods. An average sidewall roughness (Sa) of 1.205 µm is achieved when cutting a 112 µm‐thick GaAs wafer. In addition, the dicing process induces low residual stress, measured at 0.461 GPa. These results demonstrate that the proposed method is effective for cutting large, ultrathin, hard, and brittle GaAs wafers. It can help improve yield and reliability in integrated circuit (IC) chip production.

## Graphical abstract

This study presents a femtosecond laser‐based ultrashort pulse dicing system that uses a non‐diffracting Bessel beam to shape the femtosecond laser. The use of femtosecond Bessel beam shaping and high‐speed scanning enables rapid material removal that outpaces thermal diffusion, thereby suppressing melting, minimizing the HAZ, and reducing sidewall roughness.

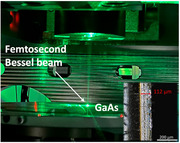

## 1 Introduction

III‐V semiconductors, such as gallium arsenide (GaAs), are widely used due to their superior electronic properties compared to silicon (Si). In particular, GaAs offers a low‐temperature coefficient and high optical absorption, making it suitable for applications with limited surface area, such as aircraft, automobiles, and small satellites.^[^ ^1^ ^]^ GaAs‐based devices, including solar cells,^[^ ^2^, ^3^ ^]^ terahertz photonics,^[^ ^4^, ^5^ ^]^ and passive circuits,^[^ ^6^, ^7^ ^]^ benefit from these characteristics.

Despite its advantages, GaAs is a hard, and brittle material, which makes its ultra thin wafer dicing a major bottleneck in the production of GaAs‐based integrated circuits (ICs).^[^ ^8^ ^]^ Poor dicing quality reduces production yield and increases manufacturing cost. In semiconductor packaging, wafers must be separated into individual dies after front‐end processing.^[^ ^9^ ^]^ The quality of the dicing step directly impacts chip performance and yield. The most common method is mechanical dicing with a diamond blade,^[^ ^10^ ^]^ but this approach generates high chipping rates and is unsuitable for hard and brittle materials such as SiC^[^ ^11^ ^]^ and GaAs.^[^ ^12^ ^]^

In recent years, laser‐based dicing techniques—especially those using nanosecond and picosecond pulses—have emerged as promising alternatives.^[^ ^13^, ^14^ ^]^ Among them, laser stealth dicing has shown success in materials like silicon (Si),^[^ ^15^ ^]^ SiC,^[^ ^16^ ^]^ and transparent substrates, including lithium tantalate (LiTaO_3_), lithium niobate (LiNbO_3_), and Ce: YAG.^[^ ^17^ ^]^ However, stealth dicing is not applicable to GaAs due to its ultra‐thin structure and optical properties.

Alternatively, full‐thickness cutting using water jet‐guided lasers has been attempted,^[^ ^18^, ^19^ ^]^ but the brittle nature of GaAs causes severe chipping and leads to a rough cut surface. Nanosecond and picosecond laser full‐thickness cutting methods have also been explored,^[^ ^20^ ^]^ but they still result in high chipping rates and increased surface roughness when applied to large‐size, ultra‐thin GaAs wafers (see Figure S1, Supporting Information). Therefore, an advanced dicing solution is required to achieve chipping‐free edges, low kerf width loss, and minimal surface roughness, thereby improving production yield and reducing cost in GaAs‐based chip manufacturing.

In this study, we performed full‐thickness cutting experiments on 112 µm‐thick GaAs wafers using a femtosecond (fs) Bessel laser beam combined with an XY‐galvanometer scanner. The method achieved no edge chipping, a high scanning speed of 2000 mm·s^−1^, and a kerf width loss below 17 µm. The average surface roughness (Sa) in the cutting sidewall was measured at 1.205 µm. These results demonstrate that fs laser dicing method is a promising technique for processing large‐size, ultra‐thin, hard, and brittle GaAs wafers, offering improved yield and quality in IC chip manufacturing.

## 2 Results and Discussion

### 2.1 The Interaction Between Ultrafast Laser Pulses and GaAs wafers

**Figure 1:**
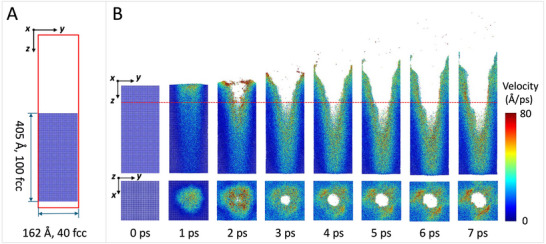
The interaction between ultrafast laser pulses and GaAs wafers. A) The simulation model size for GaAs. B) The molecular dynamics simulation of the interaction between the laser and GaAs.

To investigate the interaction between ultrafast laser pulses and GaAs at the atomic scale, we conducted molecular dynamics (MD) simulations. **Figure** **1**A presents the simulation model, consisting of a vacuum region and a GaAs crystal containing 1024000 atoms, with initial dimensions of L*z* = 452.272 Å and L*x* = L*y* = 226.136 Å. The *x*‐axis of the model is aligned with the [0, 0, 1] crystallographic direction of GaAs. Ultrafast laser pulses were applied from the positive *z*‐direction, targeting the surface of the GaAs crystal. All simulations were performed using the LAMMPS package with the Velocity‐Verlet algorithm. The system was first relaxed under the NVT ensemble at 300 K, and then irradiated with laser pulses having a power density of 1.602E14 W/cm^2^, a pulse duration of 470 fs, and an incident depth of 162 Å.

During the interaction between the laser and GaAs, the laser energy is primarily absorbed by free electrons via the inverse bremsstrahlung effect.^[^ ^21^ ^]^ As GaAs is a direct bandgap semiconductor with a bandgap energy of ~1.42 eV at room temperature, a laser wavelength (λ) of 515 nm was selected. The photon energy at this wavelength is 2.14 eV, as calculated by the equation *E* = *hc*/λ. This energy is sufficient to exceed GaAs's bandgap, ensuring efficient energy deposition in a microscopic surface volume.

After absorption, thermalization occurs within the electron subsystem. In this process, energy is transferred from the electrons to the lattice through electron–phonon coupling, and heat is subsequently transported by electron thermal diffusion. The lattice heating time typically occurs on the picosecond timescale. Melting takes place when the laser pulse duration exceeds the thermal relaxation time of GaAs. During the subsequent melt–cooling cycle, residual stress develops, which can lead to edge chipping. To mitigate thermal accumulation during GaAs wafer dicing, a pulse duration shorter than the thermal relaxation time was selected. Therefore, a green‐light femtosecond laser with a 470 fs pulse duration and a scan speed of 2000 mm·s^−1^ was employed.

Figure 1B presents the structural evolution of GaAs irradiated by ultrafast laser pulses at various time steps. The top panels show longitudinal views, while the bottom panels display transverse views. Atoms are color‐coded according to their velocity magnitude. Upon laser pulse irradiation, the Ga and As atoms absorb energy and exhibit intensified thermal motion, resulting in a sharp increase in atomic velocity. As some atoms gain sufficient energy, they escape their original lattice sites, leading to lattice disorder and deformation. At 2 ps, a phase transition is observed: the center of the laser‐irradiated region begins to ablate, forming a V‐shaped crater, which indicates that the local temperature exceeds the melting point of GaAs. The ablated atoms are rapidly ejected into the vacuum region. As laser energy accumulates, the depth and radius of the ablation crater increase progressively. By 7 ps, the process reaches the preset penetration depth of 162 Å. Notably, no significant melt pool is observed during the interaction. This suggests that thermal accumulation is effectively suppressed due to the ultrashort pulse duration of 470 fs, which is shorter than the characteristic lattice heating time on the picosecond scale. As a result, thermalization within the electron subsystem is terminated before energy fully transfers to the lattice. These simulations visually demonstrate the dominance of non‐thermal ablation processes over thermal melting at the femtosecond timescale.

The MD simulation results unravel the fundamental ultra‐fast physics governing the initial single‐pulse interaction between the femtosecond laser and the GaAs lattice. Moreover, the results show clean material removal with minimal residual damage below a specific fluence threshold, thereby providing the theoretical support to pursue high‐power, high‐repetition‐rate experimental parameters. Thus, even at high average powers, each individual pulse can still interact with the GaAs in a predominantly non‐thermal regime, preventing catastrophic thermal accumulation.

### 2.2 Analysis of Different Processes on GaAs Wafer Dicing

**Figure 2:**
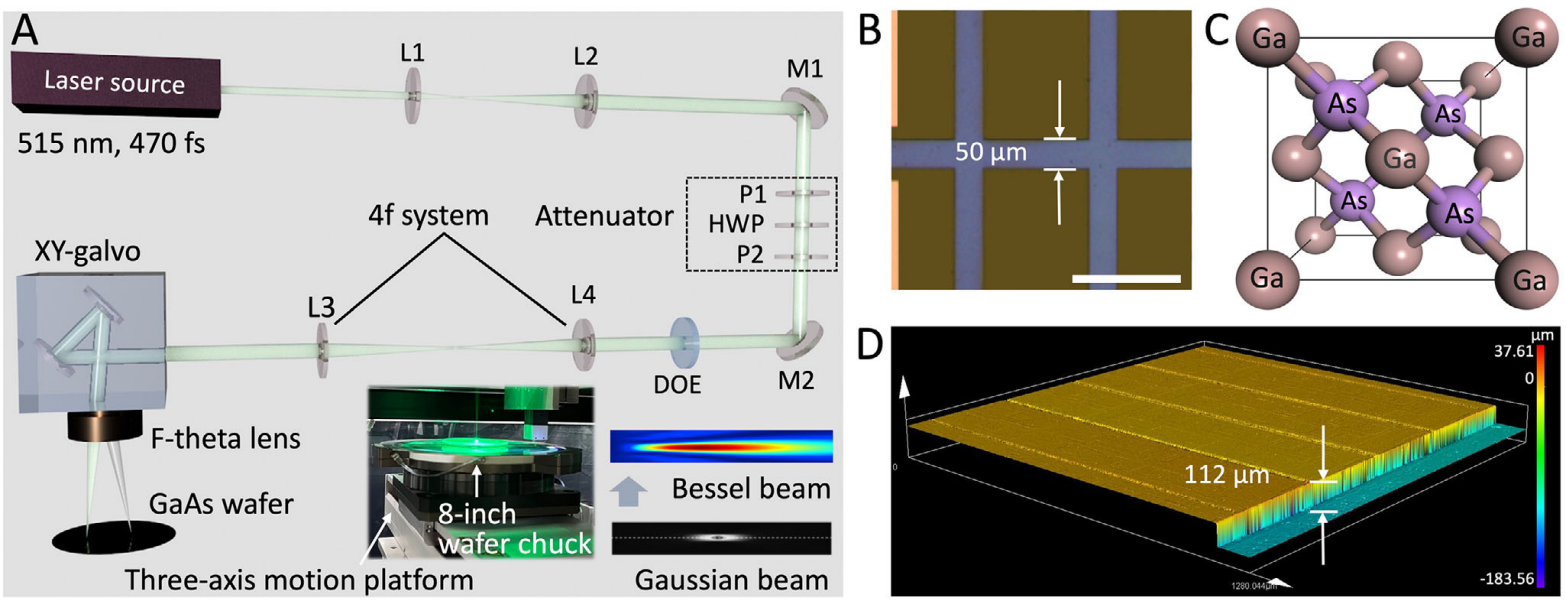
Schematic illustrations of the femtosecond laser‐based GaAs dicing system and the schematic drawing of the GaAs wafer. A) Experimental schematic of the femtosecond laser system and optical setup for GaAs wafer dicing using an *f*‐theta lens. P1 and P2: polarizers, L1–L4: lenses, HWP: half‐wave plate. Inset: wafer chuck for securing an 8‐inch GaAs wafer during processing. B) The width of the dicing street between dies is 50 µm, and the scale bar is 200 µm. C) The crystal structure of the GaAs cubic unit cell, D) Wafer thickness measured as 112 µm using LSCM.

**Figure** **2**A shows the experimental setup used for GaAs wafer dicing, which consists of a femtosecond laser source, a diffractive optical element (DOE), an XY‐galvanometric scanner, an *f*‐theta lens, and a three‐axis motorized stage. Ultrafast laser pulses with a duration of 470 fs and a wavelength of 515 nm were generated by a femtosecond laser system. The laser provided an average power of 40 W with a tunable repetition rate from 200 to 2000 kHz. At 200 kHz, the pulse energy reached 200 µJ. A linearly polarized laser beam was directed through a variable attenuator to adjust the pulse energy. The collimated beam, with a Gaussian profile, was converted into a Bessel beam using a DOE. An iterative optimization algorithm (Gerchberg–Saxton) was used to calculate the DOE's phase profile, with the sidelobe suppression incorporated as a direct constraint in the merit function (cost function) that the algorithm minimizes. This means the algorithm was explicitly tasked with finding a phase solution that not only forms a central core but also penalizes high intensity in the sidelobe regions. The Bessel beam was then focused through an *f*‐theta lens with an effective focal length of 67.2 mm.

Figure S2A (Supporting Information) shows the extended focus effect in the normalized XZ intensity profile, obtained through Zemax simulation. The calculated diffraction‐limited spot size (the beam waist) was 7.32 µm, and the depth of field (DOF) in air was 5.834 mm (Figure S2B, Supporting Information). Figure S2C (Supporting Information) shows the transverse beam pattern at the focus obtained at different focus lengths on the GaAs wafer surface. The beam waist and depth of field values presented (7.5 µm and 6 mm, respectively) are in excellent agreement with the theoretical values calculated for a Bessel beam under our experimental conditions.

The fusion of femtosecond lasers with Bessel beams creates a premier “cold” processing tool, uniquely enabling high‐aspect‐ratio ablation deep within materials, as the Bessel beam's long focal zone confines the ultrafast laser's energy precisely along its propagation path.^[^ ^22^ ^]^ This combination ensures exceptional processing quality—characterized by minimal heat damage, negligible sidewall roughness, and low residual stress—while simultaneously offering remarkable resilience to focal positioning errors, making it ideal for dicing brittle wafers. Figure S3 (Supporting Information) shows the kerf width and cross‐sectional profiles resulting from Gaussian and Bessel beams under identical processing conditions. The results demonstrate that the Bessel beam achieves a superior aspect ratio, attributable to its long depth of field and non‐diffracting nature. This enables the creation of deep, narrow grooves unattainable with a diffracting Gaussian beam. Furthermore, the Bessel beam is insensitive to surface topography as it maintains a consistent spot size and intensity even on non‐flat wafer surfaces.

An XY‐galvanometric scanner was used for beam steering, with a maximum deflection speed of 30 rad·s^−1^. Combined with the *f*‐theta lens, a scanning speed of 2000 mm·s^−1^ was achieved. For all tested repetition rates between 600 and 2000 kHz, a constant scanning speed of 2000 mm·s^−1^ was used to maximize process efficiency. During operation, the focused beam was continuously scanned over the wafer surface via the galvanometric mirrors, while the Z‐axis movement was controlled by the motorized stage. The scanner's two‐axis deflection system steered the beam across the X‐Y plane, providing a scan field of 15  ×  15 mm for precise 2D patterning. In addition, a three‐axis stage with a positioning accuracy of less than 1 µm and a range of 400 mm was used to stitch the scan fields of the galvanometer scanners, enabling the system to handle an 8‐inch GaAs wafer. As shown in the inset of Figure 2A, the GaAs wafer (8‐inch diameter) was mounted on a wafer chuck fixed to the three‐axis stage for alignment and stability during laser processing.

GaAs wafer samples with predefined die dimensions and dicing streets were prepared for laser processing. Figure 2B illustrates the wafer layout and its structural parameters, the width of the dicing street between adjacent dies is 50 µm. Figure 2C shows the lattice structure of GaAs crystal, commonly known as the zincblende structure, with a lattice constant of 5.6534 Å. The GaAs crystal consists of two face‐centered cubic (FCC) sublattices—one of gallium (Ga) and the other of arsenic (As). Ga atoms are represented in rosy‐brown, while the As atoms are shown in purple. Each As atom is surrounded by four Ga atoms, forming a tetrahedral bond similar to that in a diamond lattice, but composed of different elements. This structure results in ionic bonding due to the presence of two distinct atomic species.^[^ ^23^ ^]^ Figure 2D presents the wafer thickness, which was measured to be 112 µm using laser scanning confocal microscopy (LSCM).

**Figure 3:**
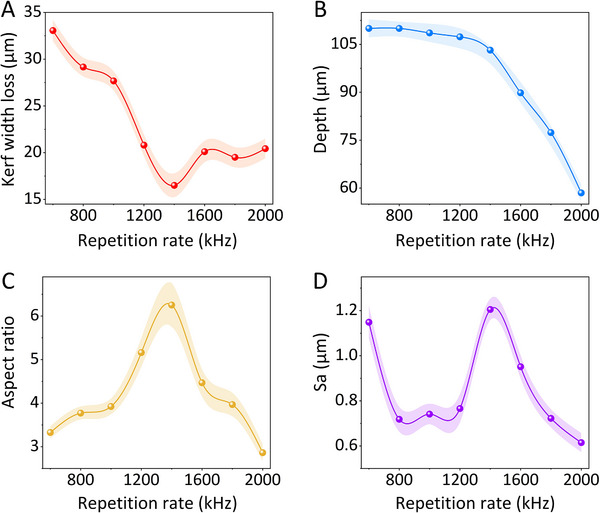
A) Kerf width, B) cutting depth, C) aspect ratio, and D) average surface roughness of the diced GaAs wafers processed by a femtosecond laser with a repetition rate range from 600 to 2000 kHz.

**Figure** **3**A shows the variation in kerf width at different repetition rates ranging from 600 to 2000 kHz, under an average laser power of 5 W. The number of laser scans is 30. The corresponding spot overlap spans from 54.47% to 86.33%. This translates to a spatial pulse separation of 1 to 3.33 µm. Because the time interval between pulses arriving at adjacent points is shorter than the material's heat dissipation time. Residual thermal energy from previous pulses accumulates, thereby raising the local temperature. This accumulated heat significantly lowers the effective ablation threshold for subsequent pulses, leading to more efficient material removal and deeper/wider grooves.

As the repetition rate increases, the kerf width narrows and stabilizes beyond 1200 kHz, maintaining values between 16 and 20 µm. Figure 3B presents the change in cutting depth, ranging from 55 to 112 µm across the same repetition rate range. Due to the inverse relationship between pulse energy and repetition rate, the deepest cut (112 µm) occurs at 600 kHz, sufficient to penetrate typical GaAs wafer thicknesses used in IC fabrication. Figure 3C shows the aspect ratio of the diced GaAs dies. The aspect ratio significantly increases with repetition rate and reaches a maximum value of 6.25 at 1400 kHz, corresponding to an 80.48% spot overlap. This indicates that kerf width loss can be kept below 20 µm when processing 100 µm‐thick GaAs wafers. Measuring the sidewall roughness is typically challenging in the dicing of full‐thickness due to edge chipping. However, in this study, the high cutting quality achieved with femtosecond laser dicing allowed reliable roughness measurements. As shown in Figure 3D, the average sidewall roughness (Sa) remains below 1.205 µm across all tested repetition rates. Notably, no edge chipping was observed on any of the processed GaAs wafers.

### 2.3 Analysis of Chipping, Roughness, and Residual Stress on the Diced GaAs Die

The shorter the laser pulse width, the higher the dicing precision. This is due to a fundamental shift in the material removal mechanism. Long pulses (nanoseconds, ns) cause significant thermal diffusion, leading to melting, large HAZ, recast layers, and micro‐cracks. This limits precision and causes collateral thermal damage. In contrast, short pulses (picoseconds, ps) reduce thermal effects significantly. Material is removed through a mix of thermal and non‐thermal processes (e.g., phase explosion), resulting in much finer features and less damage. Finally, ultra‐short pulses (femtoseconds, fs) enable truly non‐thermal “cold” ablation. Energy is deposited faster than heat can diffuse away. Material is directly vaporized via multi‐photon ionization with virtually no heat‐affected zone, allowing for sub‐micron, high‐quality machining with minimal debris and extreme precision. In essence, shorter pulses confine energy more precisely to the laser focus, eliminating the thermal effects that degrade accuracy.

**Figure 4:**
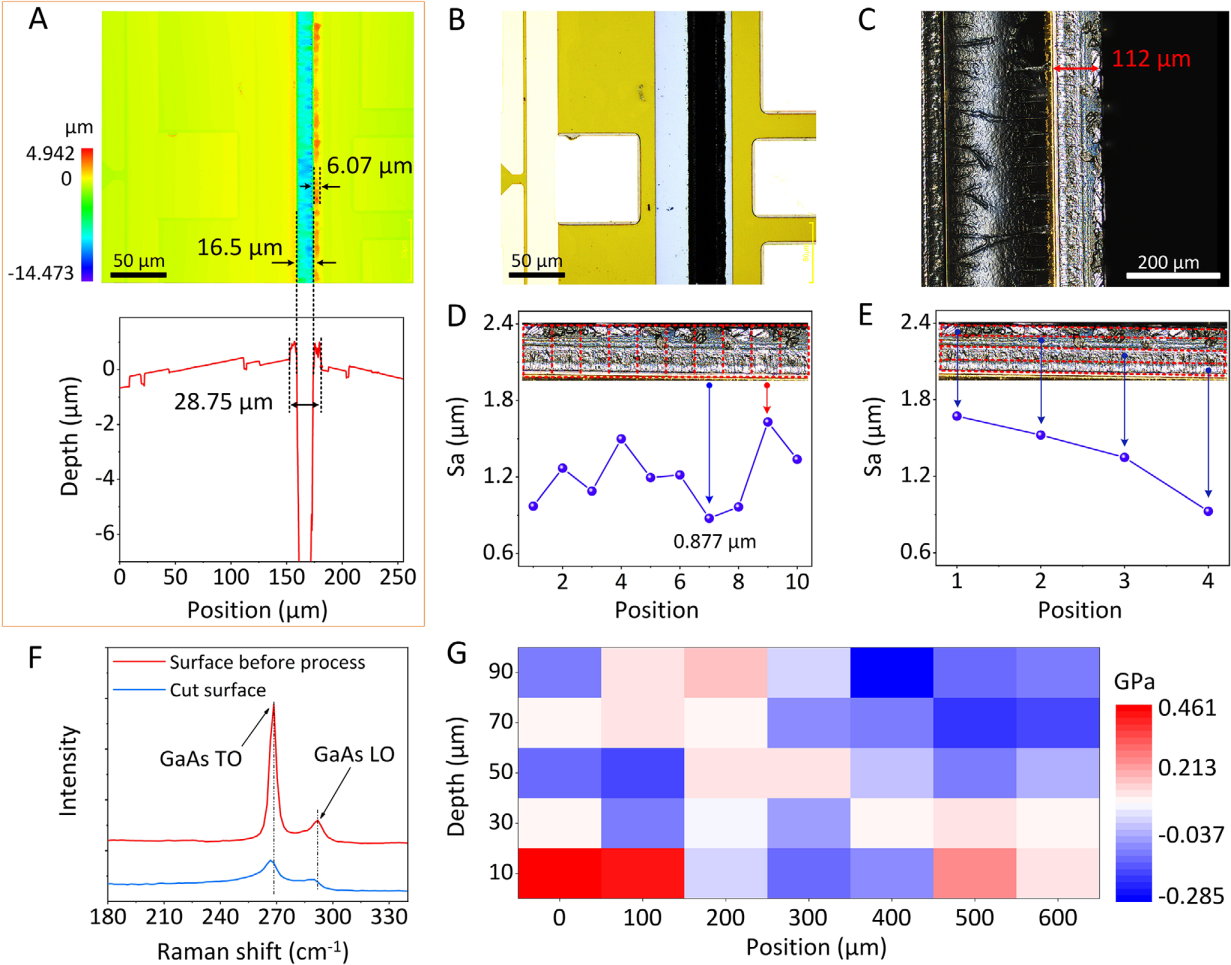
A) Surface morphology of the diced GaAs wafer processed with a femtosecond laser (wavelength: 515 nm, power: 5 W, repetition rate: 1400 kHz), showing a HAZ of 6.07 µm, and a kerf width of 16.5 µm. B) Top view of the cutting street on the wafer surface. C) Cross‐sectional image of the cutting section after dicing. Spatial variation of the average surface roughness (Sa) across different D) regions and E) depths of the cutting sidewall. F) Raman spectra from the laser‐processed surface (black curve), showing GaAs peaks: transverse optical (TO) at 266.729 cm^−1^ and longitudinal optical (LO) at 290.145 cm^−1^. G) Residual stress mapping on the cut surface.

Figure S4 (Supporting Information) compares the dicing performance of picosecond and femtosecond pulses. The quality of laser‐based dicing processes is evaluated based on critical parameters such as chipping and the HAZ. As the Figure illustrates, the chipping and HAZ formation near the kerf when a picosecond laser with a 7 ps pulse duration was used to dice a GaAs wafer. As shown, the chipping width was 12.1 µm, and the HAZ width reached 14.5 µm, about 1.4 times the kerf width (10.3 µm). In contrast, **Figure** **4**A shows that no chipping occurred at the kerf edge when dicing GaAs with a femtosecond laser having a 470 fs pulse duration and a 1400 kHz repetition rate. This is attributed to the absence of heat melting and minimal residual stress. The observed HAZ width was only 6.07 µm, about one‐third of the kerf width (16.5 µm). Compared to the ps‐laser case, the HAZ‐to‐kerf width ratio was significantly reduced with fs‐laser dicing, down to 0.238 ×.

Figure 4B,C present the top‐view dicing streets and cross‐sectional profile, respectively. A cutting depth of 112 µm was achieved, matching the full wafer thickness, thereby confirming that full‐thickness cutting of brittle GaAs wafers can be realized using a femtosecond laser system. Figure 4D shows spatial variations in the average sidewall roughness (Sa) across different regions of the cut section under 1400 kHz. As indicated by the blue arrow, the minimum roughness was 0.877 µm, while the maximum roughness, shown by the red arrow, was 1.338 µm. Additionally, as shown in Figure 4E, the Sa value decreases with increasing depth. This trend can be attributed to greater heat accumulation near the top surface of the GaAs wafer, where the laser beam initially interacts with the material. Subsequent pulses impact areas that have been preheated by previous pulses. This progressive buildup of heat leads to more pronounced thermal effects, such as micro‐melting and redeposition, resulting in a relatively higher surface roughness. As processing progresses to greater depths (toward the bottom of the GaAs wafer), material is removed, and heat can dissipate more easily into the surrounding bulk material. Furthermore, as some laser energy is dissipated while penetrating the material, the heat accumulation effect is significantly reduced at the bottom. Consequently, the ablation mechanism more closely resembles the ideal “cold” ablation, yielding a lower surface roughness.

Investigating the mechanical behavior of diced GaAs is essential, as mechanical failure is a primary failure mode in GaAs‐based devices. However, traditional dicing studies have rarely addressed mechanical performance. In particular, the impact of residual stress on mechanical integrity has been largely overlooked, due to the difficulty of characterization in brittle materials like GaAs.

Residual stress significantly affects the performance of GaAs devices, influencing properties such as carrier mobility and luminescence efficiency.^[^ ^24^, ^25^ ^]^ As a nondestructive technique, Raman spectroscopy has been widely used to evaluate surface crystalline quality and interfacial strain in heterostructures.^[^ ^26^, ^27^, ^28^ ^]^ In this study, residual biaxial strain at the cross‐section of the diced GaAs wafer was characterized using Raman spectra to assess the structural quality following femtosecond laser dicing.

The Raman spectra are shown in Figure 4F. In the low‐frequency range from 200 to 340 cm^−1^, distinct peaks corresponding to transverse optical (TO) and longitudinal optical (LO) phonon modes are observed, which are commonly used as indicators of residual stress. The red curve represents the Raman spectrum of the pristine GaAs wafer surface before dicing. In contrast, the black curve corresponds to the cut surface after laser processing. A blue shift in both TO and LO peaks is observed in the cut surface compared to the pristine wafer, indicating a lattice disturbance and the introduction of residual stress due to the laser dicing process.

As shown in **Table** **1**, the TO and LO phonon modes of the pristine GaAs surface appear at 268.553 and 291.944 cm^−1^, respectively. After laser dicing, these modes exhibit a blue shift, with the corresponding values at the cut section measured as 266.729 cm^−1^ (TO) and 290.145 cm^−1^ (LO). This spectral shift indicates the presence of residual tensile stress, which alters the lattice vibration frequencies. The residual stress (σ, in GPa) can be estimated from the Raman peak shift of the LO mode (Δω, in cm^−1^) using the following relation:^[^ ^29^, ^30^ ^]^

(1) $$\Delta \omega_{L}=-3.9\sigma$$

where Δω_L_ is the observed shift of the LO photon mode. For a measured shift of Δω_L_ = −1.799 cm^−1^ (calculated from 291.944 − 290.145 cm^−1^), the residual tensile stress introduced by the laser dicing process is estimated to be 0.461 GPa.

**Table 1:** The data of the TO and LO modes.

	Surface before process	Cut surface
Raman TO (cm^−1^)	268.553	266.729
Raman LO (cm^−1^)	291.944	290.145

Figure 4G presents the residual stress distribution on the cut surface. The maximum tensile stress was 0.461 GPa, while the maximum compressive stress was 0.285 GPa. To investigate the mechanism by which the cutting process influences the material, hardness mapping was performed via nanoindentation (Figure S5, Supporting Information). The region of compressive stress (denoted by the black box in Figure 4G) correlates with an increase in hardness in the same area (Figure S5, Supporting Information). As compressive stress increases, the dislocation density rises sharply. The moving dislocations interact with other dislocations, grain boundaries, and secondary obstacles, resulting in entanglement and pile‐up. These interactions hinder the subsequent movement of dislocations. To continue deformation, a greater stress is required. Macroscopically, this phenomenon manifests as an increase in GaAs's hardness.

In **Table** **2**, residual stress is commonly introduced by various wafer processing methods, such as blade dicing, laser cutting, and chemical vapor deposition (CVD). These stresses often arise from thermal gradients, mechanical forces, or phase transformations during fabrication. Notably, in the present study, the femtosecond laser‐based dicing process produced the lowest residual stress among the listed techniques. This result highlights the superior thermal confinement and non‐contact nature of ultrafast laser processing, which minimizes heat‐affected zones and stress accumulation. Therefore, this dicing method offers a promising route to improve the dicing quality and mechanical integrity of GaAs wafers. It is expected to significantly enhance the production yield of GaAs‐based chips in IC manufacturing.

**Table 2:** Residual stress of various materials induced after dicing.

	Materials	Processing method	Residual stress	Refs.
1	GaAs	By blade sawing	1.5 GPa	[31]
2	GaAs	By blade sawing	1.97–7.06 GPa	[32]
3	Si	By blade sawing	1.1 GPa	[33]
4	GaAs	By blade sawing	1.76–2.24 GPa	[34]
5	3C‐SiC	By the CVD method	1.301 GPa	[35]
6	Reaction‐bonded SiC	By irradiation	1.186 GPa	[36]
7	3C–SiC	By scratch	2.6 GPa	[37]
8	GaAs	By laser	0.461 GPa	Present work

### 2.4 Analysis of Dicing Efficiency on A 4‐inch GaAs‐based IC Wafer

**Figure 5:**
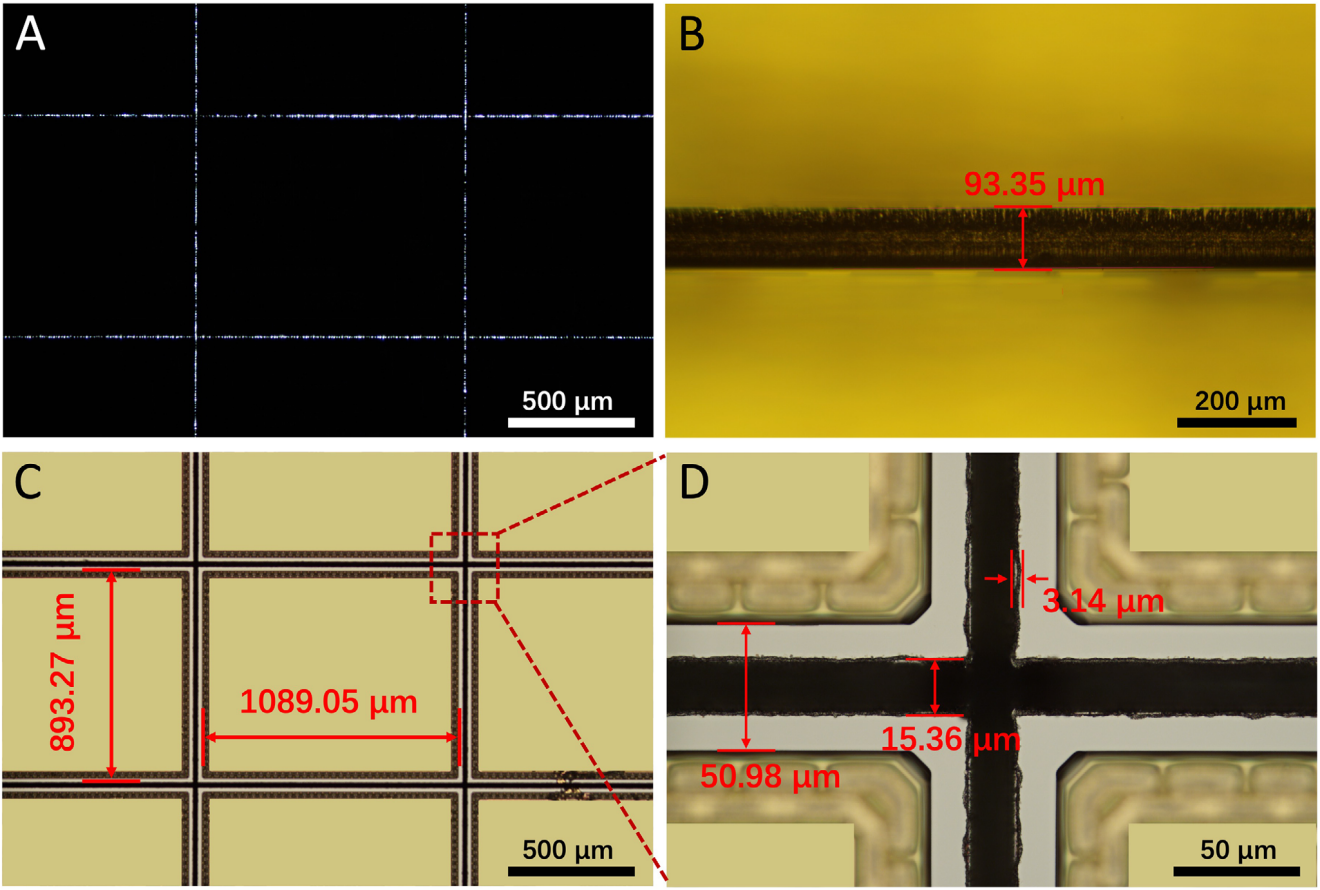
A 4‐inch GaAs‐based IC wafer is cut into individual chips. A) The shapes of each die on the back side. B) The thickness of the die is measured on the die sidewall. C) The size of each die, and D) the kerf width loss and a minimized HAZ are measured on the wafer surface.

To study the dicing efficiency of the femtosecond laser system, a 4‐inch GaAs‐based IC wafer was used (Figure S6, Supporting Information). As shown in **Figure** **5**A, the full‐thickness cutting method was employed because the die outlines were visible on the back side after processing. The resulting thickness was 93.35 µm (Figure 5B). Figure 5C presents the dimensions of a single die on the wafer surface, with a length and width of 1089.05 and 893.27 µm, respectively. The kerf width loss was 15.36 µm, and a minimal HAZ of only 3.14 µm was observed (Figure 5D).

Dicing streets comprising 89 lines in the X direction and 109 lines in the Y direction were set based on the die parameters. As shown in Figure S7 (Supporting Information), the total length of the dicing streets on the 4‐inch IC wafer was ~9146.022 mm. The total scan length was calculated as the total number of scans (25) multiplied by the total street length, resulting in ~228655.5 mm. Using the galvanometric scanner at a speed of 2000 mm·s^−1^, the total scanning time was ~114.33 s. The motion platform required an additional ~9.14 s for its travel. Therefore, the total process time was ~123.47 s, corresponding to a full‐thickness cutting speed of ~74.08 mm·s^−1^.

## 3 Conclusion

In this work, a femtosecond laser‐based ultrashort pulse dicing system was developed and optimized for the full‐thickness cutting of ultra‐thin, hard, and brittle GaAs wafers. The system—comprising a femtosecond laser, XY‐galvanometric scanner, *f*‐theta lens, and a three‐axis motion platform—achieved precise and damage‐free dicing with no observable chipping. The use of femtosecond Bessel beam shaping and high‐speed scanning enabled rapid material removal that outpaced thermal diffusion, thereby suppressing melting, minimizing the HAZ, and reducing sidewall roughness. A smooth cut surface with an average roughness (Sa) of 1.205 µm was obtained, along with a low residual tensile stress of 0.461 GPa in a 112 µm‐thick GaAs wafer processed at 1400 kHz with an 80.48% spot overlap. A 4‐inch GaAs‐based IC wafer was cut into individual chips with high efficiency in ~123.47 s, which corresponds to a full‐thickness cutting speed of ~74.08 mm·s^−1^.

Compared with traditional blade and nanosecond/picosecond laser dicing methods, this approach demonstrates superior thermal confinement and structural integrity control, enabling high production yield and wafer reliability. This femtosecond laser‐based dicing technology presents a scalable, high‐throughput, and cost‐efficient solution for GaAs wafer processing in integrated circuit (IC) manufacturing. Future research will focus on system automation, industrial scalability, and adaptation to other brittle semiconductor materials such as InP and GaN.

## 4 Experimental Section

### Materials

The GaAs wafers were purchased from Suzhou Yancai Micro‐Nano Technology Co., Ltd. (Jiangsu, China).

### Experimental Setup

The experimental setup (Figure 2A) used for GaAs wafer dicing consists of a femtosecond laser source, a DOE, an XY‐galvanometric scanner, an f‐theta lens, and a three‐axis motorized stage. Ultrafast laser pulses with a duration of 470 fs and a wavelength of 515 nm were generated by a femtosecond laser system (Superwave, Hercules‐515–40). The collimated beam, which had a Gaussian profile, was converted into a Bessel beam using a DOE (wavelength: 515 nm, required beam diameter at 1/e^2^: 4~15 mm). This beam was then focused through an f‐theta lens (SILL Optics, S4LFT4066‐292) with an effective focal length of 67.2 mm. An XY‐galvanometric scanner (Raylase, Superscan V‐15) was used for beam steering at a maximum deflection speed of 30 rad·s^−1^. A motion stage (Akribis, AUM4‐S6) was used to stitch the scan fields and had a positioning accuracy of 1 µm.

### Characterization

The GaAs wafer was cleaned with high‐pressure deionized water for 5 min, then dried under ambient conditions. The wafer was then mounted on a clean frame and secured by a wafer chuck, allowing laser irradiation to be performed on its front surface. After laser processing, the samples were cleaned again using the same procedure as in the pre‐processing step. The diced regions were examined using optical microscopy with 10×, 20×, and 50 × objectives to obtain images of the GaAs wafer cross‐sections. The kerf width, cutting depth, and morphology of the diced area were further characterized using LSCM (Olympus OLS5100). The residual stress in the laser‐processed regions was analyzed via Raman spectroscopy using a 532 nm laser. The excitation laser power was 25 mW, with a 50 µm aperture and a 20 × objective lens, yielding a spot size of 5 µm. Each spectrum was collected with an integration time of 5 s and averaged over five accumulations.

### Numerical Simulations and Calculations

The interaction between ultra‐fast laser pulses and GaAs at the atomic scale was simulated by using the commercial LAMMPS package via the Velocity‐Verlet algorithm.

## Conflict of Interest

The authors declare no conflict of interest.

## Supporting information



Supporting Information

## Data Availability

The data that support the findings of this study are available from the corresponding author upon reasonable request.
