# Supplementary material for: Damage‐Free Full‐Thickness Dicing of Ultra‐Thin GaAs Wafers Using a Femtosecond Laser with Low Residual Stress
Source: Adv Sci (Weinh). 2025 Nov 19;13(7):e15347. doi: 10.1002/advs.202515347 (PMC12866829; doi:10.1002/advs.202515347)
Supplement: Supplementary file 1 — Supporting Information [file ADVS-13-e15347-s001.docx]

Supporting Information

Damage-free full-thickness dicing of ultra-thin GaAs wafers using a femtosecond laser with low residual stress

Shunshuo Cai*, Yankang Ding, Minxia Ding, Qi Song, Zhe Zhang, Siwei Zhang, Kunpeng Zhang, Yu Hou, Song Yue, Haiyan Shi, Man Li, Wenrui Duan, and Zichen Zhang*

1. Diced GaAs wafer by nanosecond laser


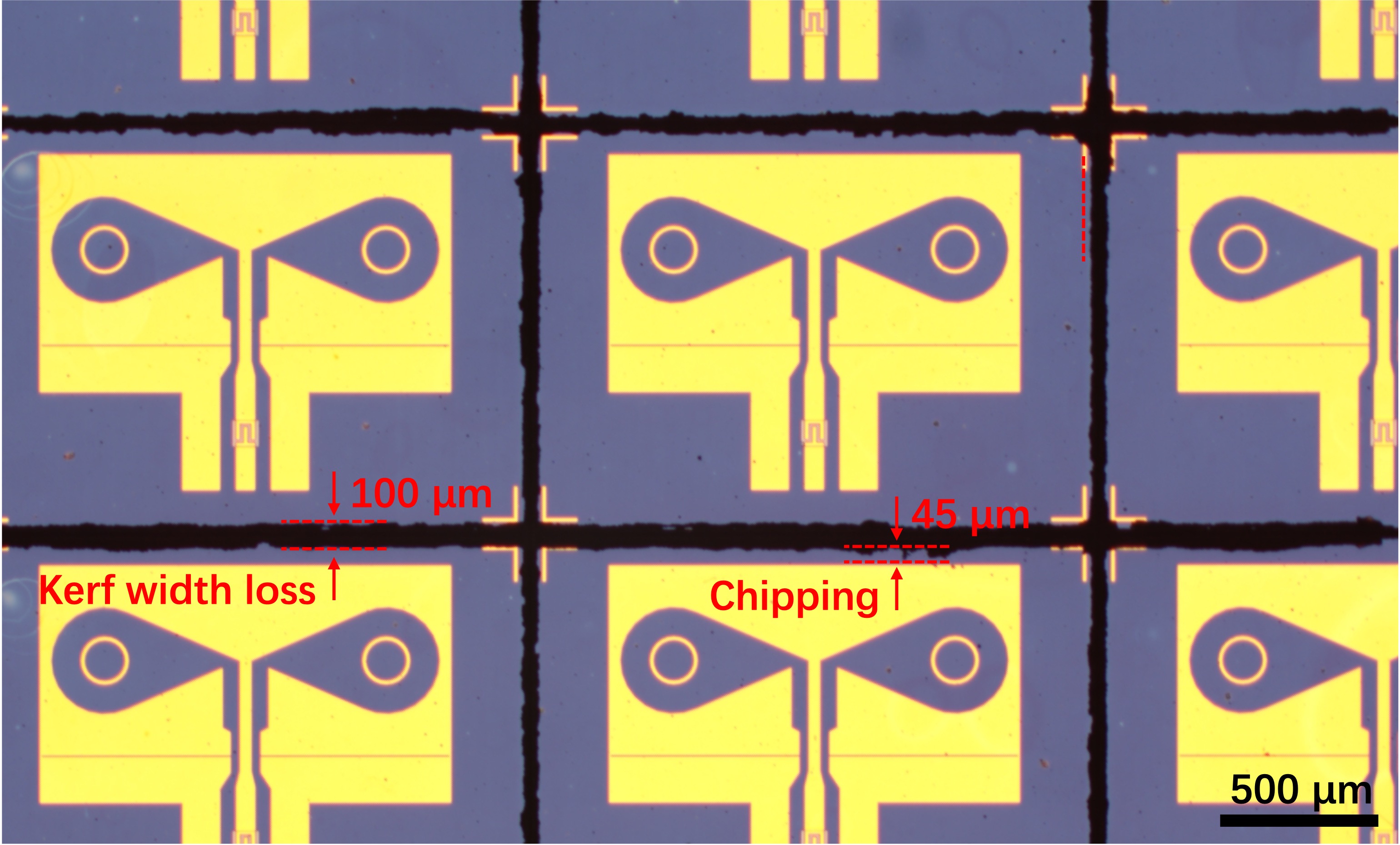


**Figure S1** The chipping, kerf width loss occur on diced GaAs wafer when using a full-thickness cutting method by a nanosecond laser.

1. Simulation of the Bessel Beam by Zemax


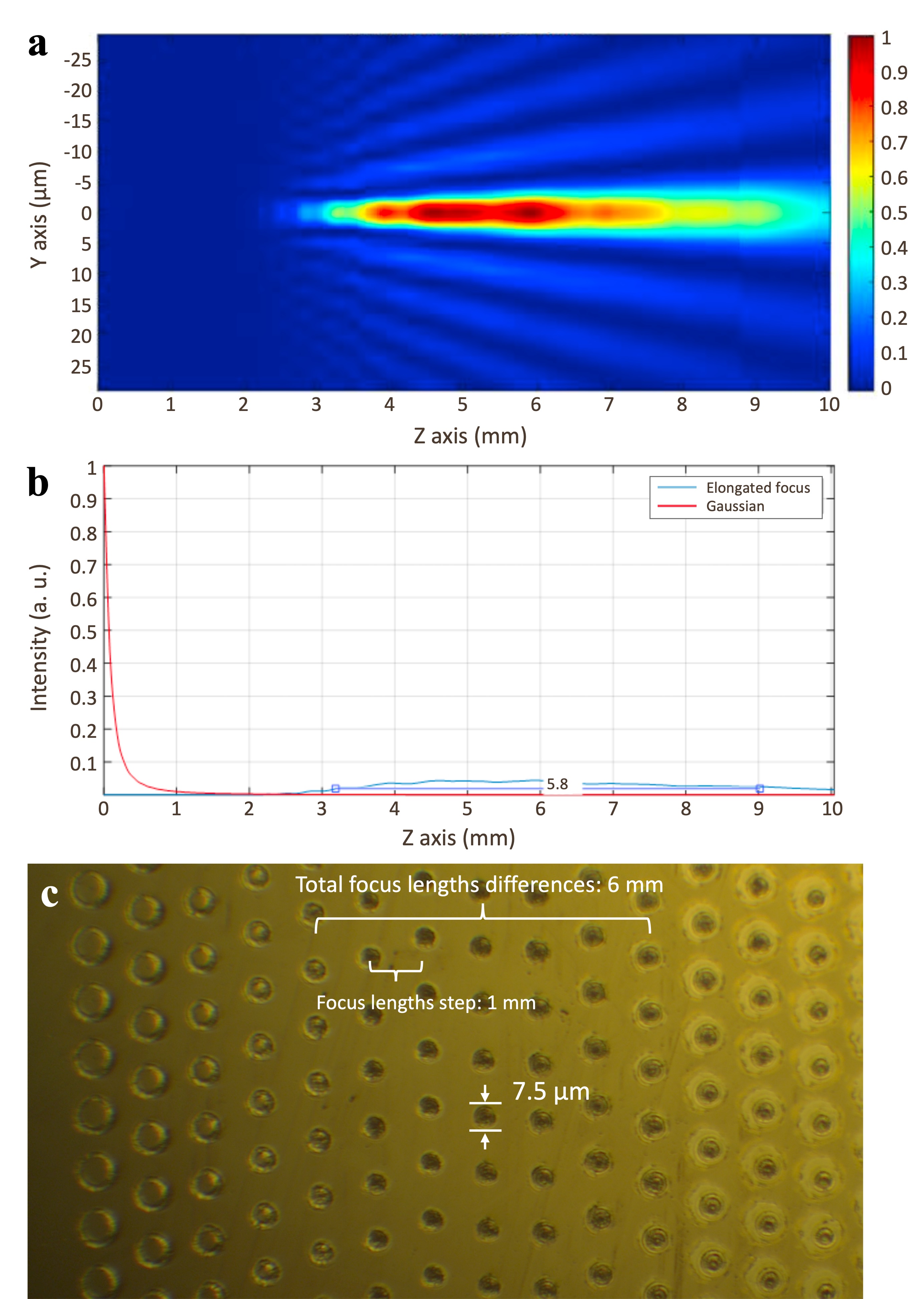


**Figure S2** (a) The extended focus effect in the normalized intensity XZ plane, (b) peak intensity normalized to Gaussian in medium with refractive index 1, (c) the transverse beam pattern at the focus obtained at different focus lengths on GaAs wafer surface.

1. The Bessel vs. Gaussian Beam Comparison


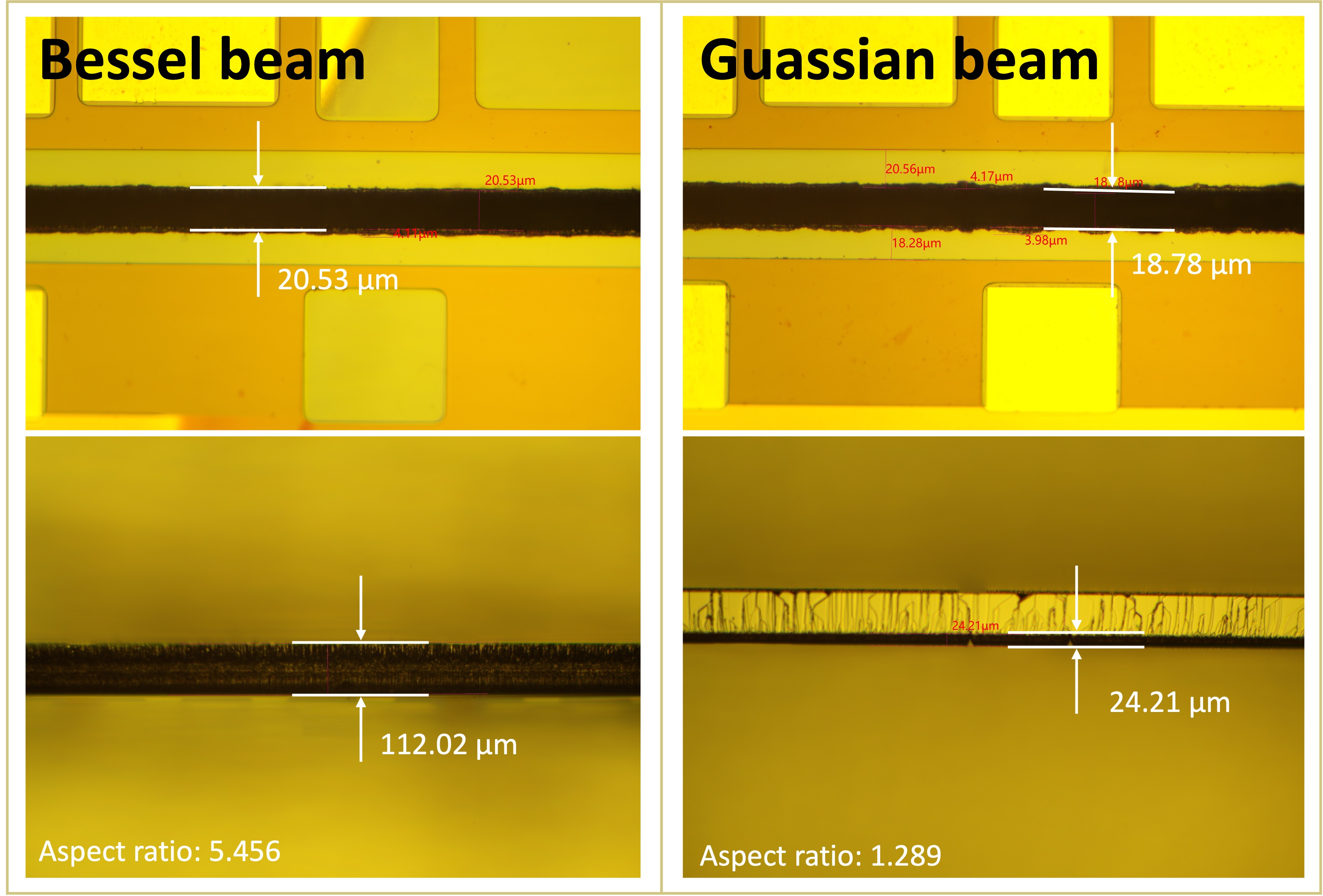


**Figure S3** Kerf loss, cross-sectional profiles machined with both Gaussian and Bessel beams, respectively, under identical processing conditions (repetition rate: 1300kHz, average laser power: 5 W, pulse width: 470 fs, scan speed: 2000 mm/s).

1. Diced GaAs wafer by picosecond pulses and femtosecond pulses.


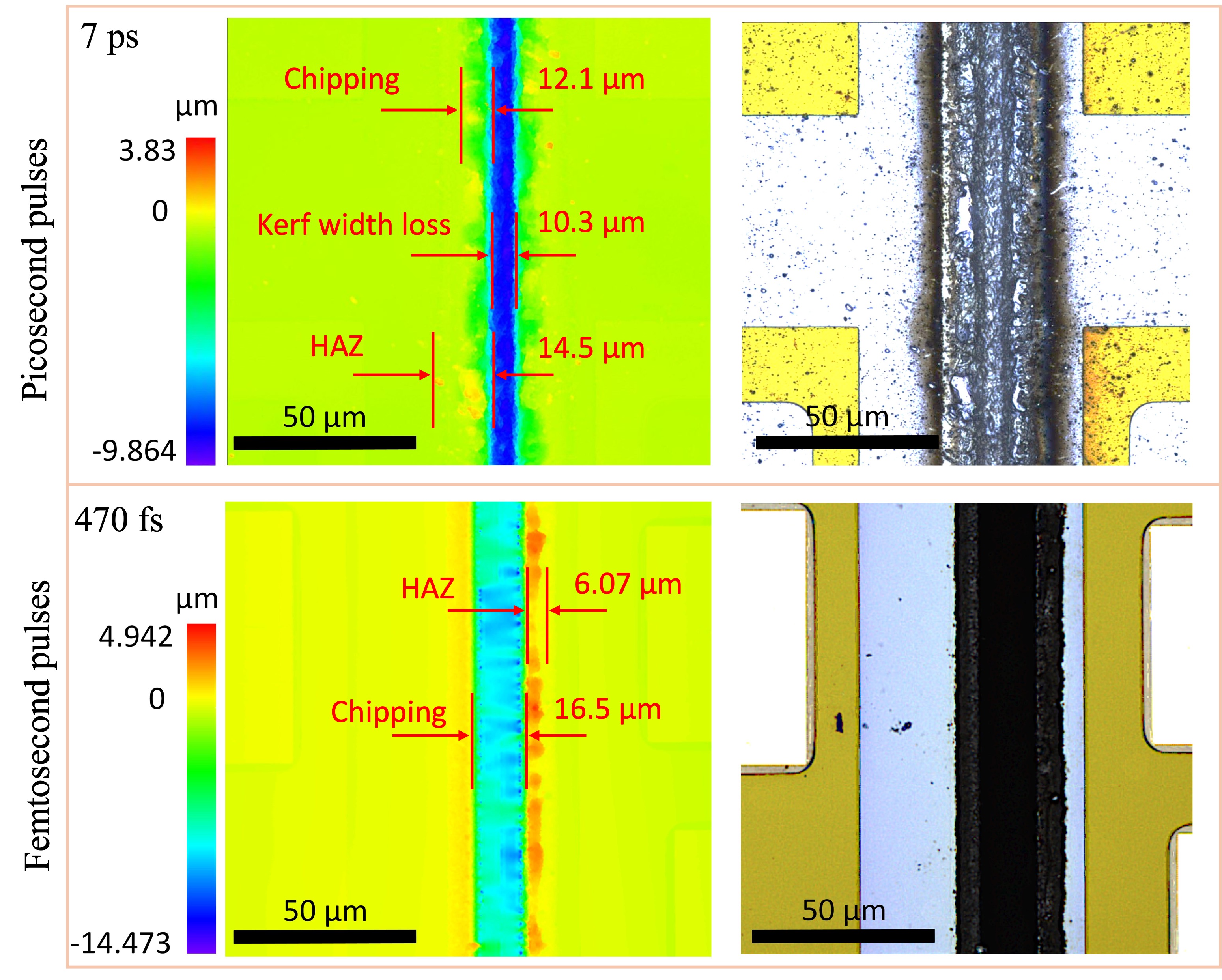


**Figure S4** A comparison of dicing performance between picosecond and femtosecond pluses. The chipping, kerf width loss, and heat area zone (HAZ) occur on a diced GaAs wafer when using a full-depth cutting method by picosecond pulses.

1. A hardness mapping of the cut surface of GaAs


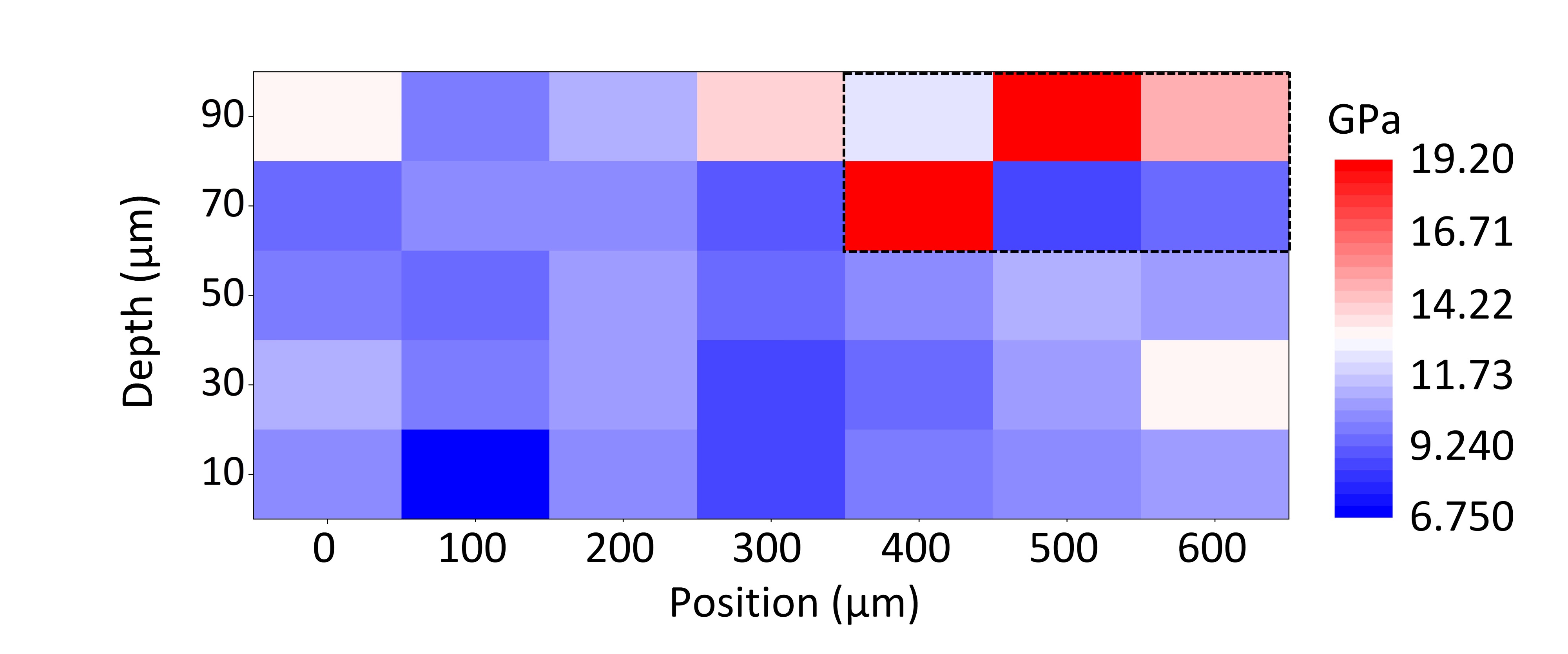


**Figure S5** A hardness mapping of the cut surface of GaAs

1. A 4-inch GaAs-based IC wafer.


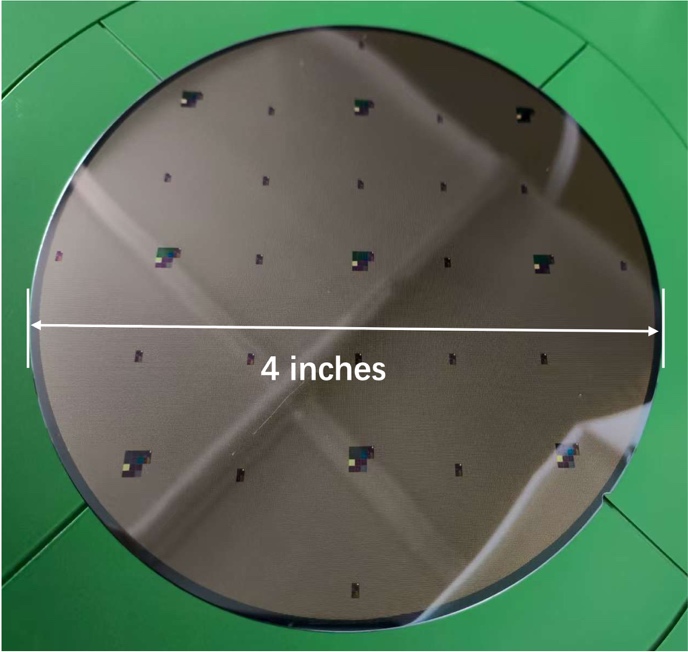


**Figure S6** A 4-inch GaAs-based IC wafer.

1. The length of the dicing streets on a 4-inch GaAs-based IC wafer.


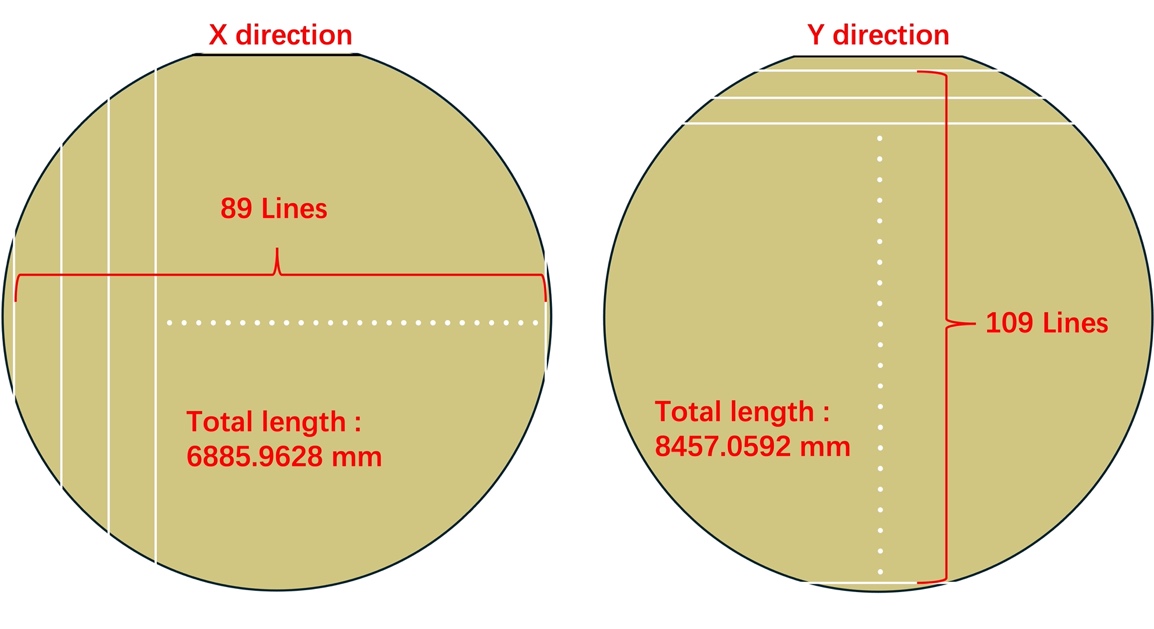


**Figure S7** The length of the dicing streets in X direction and Y direction on the 4-inch GaAs-based IC wafer.
